# Atomic-Level Characterization of the Activation Mechanism of SERCA by Calcium

**DOI:** 10.1371/journal.pone.0026936

**Published:** 2011-10-27

**Authors:** L. Michel Espinoza-Fonseca, David D. Thomas

**Affiliations:** Department of Biochemistry, Molecular Biology and Biophysics, University of Minnesota, Minneapolis, Minnesota, United States of America; Massachusetts Institute of Technology, United States of America

## Abstract

We have performed molecular dynamics (MD) simulations to elucidate, in atomic detail, the mechanism by which the sarcoplasmic reticulum Ca^2+^-ATPase (SERCA) is activated by Ca^2+^. Crystal structures suggest that activation of SERCA occurs when the cytoplasmic head-piece, in an open (E1) conformation stabilized by Ca^2+^, undergoes a large-scale open-to-closed (E1 to E2) transition that is induced by ATP binding. However, spectroscopic measurements in solution suggest that these structural states (E1 and E2) are not tightly coupled to biochemical states (defined by bound ligands); the closed E2 state predominates even in the absence of ATP, in both the presence and absence of Ca^2+^. How is this loose coupling consistent with the high efficiency of energy transduction in the Ca^2+^-ATPase? To provide insight into this question, we performed long (500 ns) all-atom MD simulations starting from the open crystal structure, including a lipid bilayer and water. In both the presence and absence of Ca^2+^, we observed a large-scale open-to-closed conformational transition within 400 ns, supporting the weak coupling between structural and biochemical states. However, upon closer inspection, it is clear that Ca^2+^ is necessary and sufficient for SERCA to reach the precise geometrical arrangement necessary for activation of ATP hydrolysis. Contrary to suggestions from crystal structures, but in agreement with solution spectroscopy, the presence of ATP is not required for this activating transition. Principal component analysis showed that Ca^2+^ reshapes the free energy landscape of SERCA to create a path between the open conformation and the activated closed conformation. Thus the malleability of the free energy landscape is essential for SERCA efficiency, ensuring that ATP hydrolysis is tightly coupled to Ca^2+^ transport. These results demonstrate the importance of real-time dynamics in the formation of catalytically competent conformations of SERCA, with broad implications for understanding enzymatic catalysis in atomic detail.

## Introduction

It has long been recognized that a large ensemble of interconverting conformations is found in the native state of a protein [1], [2]. These conformational interconversions define intrinsic protein dynamics and play essential roles in the cell, such as molecular recognition [3]–[5] and force generation [6]–[9]. Of particular interest is the mechanism by which an enzyme achieves an exquisite balance between structural heterogeneity, arising from intrinsic dynamics, and the precise geometrical arrangement required for catalysis. Considerable data from experiments and simulations have shown that conformational transitions influence catalytic activity [10]–[13], but a full atomic-level mechanism by which conformational transitions facilitate activation has not been simulated. Here, we explore in atomic detail how an enzyme, the sarcoplasmic reticulum Ca^2+^-ATPase (SERCA), selectively reaches its catalytically competent conformation.

**Figure 1:**
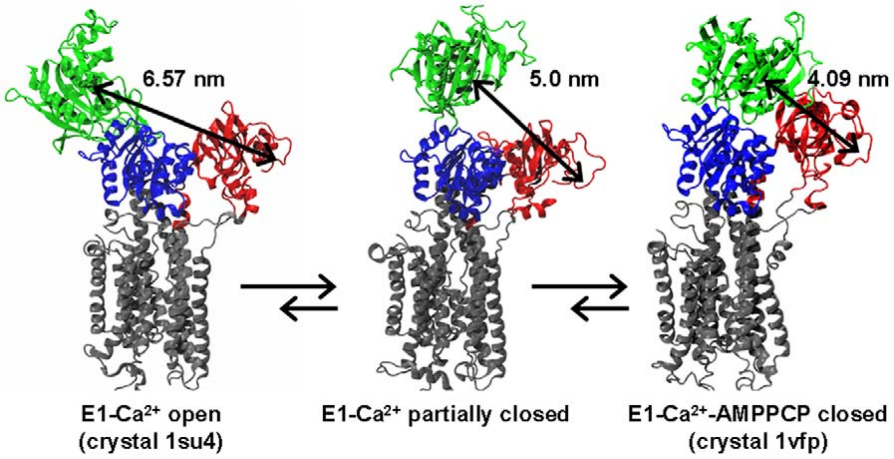
Proposed model of SERCA conformational dynamics induced by calcium. The model is based on FRET experiments (Winters et al. [16]) showing that, in the presence of calcium, the SERCA headpiece samples a broad spectrum of conformations ranging from an open (E1-Ca^2+^ open, PDB:1su4) to a fully closed one (E1-Ca^2+^-AMPPCP closed, PDB:1vfp). SERCA is colored according to its four functional domains: N domain (green), P domain (blue), A domain (red) and TM domain (grey). We highlight the C_α_-C_α_ distance between residues Met1 (A domain) and Lys515 (N domain), which were used as labeling sites to evaluate N-A interdomain distances.

SERCA, a member of the P-type ATPase family, is an integral membrane protein responsible for the active transport of Ca^2+^ from the cytoplasm into the sarcoplasmic reticulum lumen of muscle cells. Closely related SERCA isoforms are also responsible for pumping Ca^2+^ into the endoplasmic reticulum of virtually all non-muscle cells, potentiating a myriad of Ca^2+^-dependent cellular activation processes. Structurally, SERCA is formed by four functional domains: nucleotide-binding (N), phosphorylation (P), actuator (A) and transmembrane (TM) [14] (**Fig. 1**). It has been established that each catalytic cycle starts with the binding of two Ca^2+^ ions and one molecule of ATP to spatially distant sites in SERCA. The Ca^2+^-binding sites are located within the TM domain, while the N domain contains the ATP-binding site. The key step in catalysis is a reorganization of the N, P and A domains that positions the γ phosphate of ATP near Asp351 (P domain), facilitating the formation of the phosphoenzyme intermediate [15].

In the first high-resolution crystal structure of SERCA, which included bound calcium, the cytosolic headpiece (formed by the N, P and A domains) is arranged in an open conformation, with the ATP-binding and phosphorylation sites separated by more than 2.5 nm [14] (**Fig. 1**, left). Subsequently, the structure of SERCA bound to calcium and an ATP analog, adenosine 5′ -[β,γ-methylene] triphosphate (AMPPCP), showed a closed headpiece conformation favorable for ATP hydrolysis [15] (**Fig. 1**, right). Together, this crystallographic evidence suggested that (a) calcium produces a predominately open conformation, and (b) ATP binding is indispensable for the formation of the closed headpiece conformation that is necessary for calcium occlusion and phosphoenzyme formation. However, fluorescence resonance energy transfer (FRET) experiments on fully hydrated membranes in aqueous solution suggested that the cytosolic headpiece of Ca^2+^-bound SERCA is quite flexible, populating open, partially closed and closed conformations (**Fig. 1**), with the closed conformation predominating [16].

Furthermore, biochemical characterization of the effect of calcium and ATP in the activation of SERCA showed that ATP is hydrolyzed only in the presence of Ca^2+^ [17]. FRET experiments corroborated these observations, showing that calcium plays a central role in the conformational rearrangement of the cytosolic headpiece necessary for SERCA activation [18]. Other biochemical studies demonstrated that the transition of SERCA from a low-Ca^2+^-affinity conformation (E2) to a high-Ca^2+^-affinity one (E1) is not driven by Ca^2+^, but by the ionization of residues Glu309 and Glu771 of the Ca^2+^-binding site; yet in the absence of Ca^2+^, SERCA cannot undergo the conformational transitions required for catalytic activation [19].

Despite the wealth of experimental data, major issues need to be resolved at the atomic level: Does the crystal structure of Ca^2+^-bound SERCA in the open geometry represent a functional conformation? Does Ca^2+^-induced activation originate from conformational selection or an induced fit mechanism? Why is calcium binding so indispensable for SERCA activation, especially for ATP hydrolysis and phosphate transfer to SERCA? To address these questions, we have performed all-atom MD simulations of SERCA in the presence and absence of calcium, starting from the E1 state crystal structure of SERCA in the open conformation.

## Results

### Structural dynamics of Ca^2+^-bound and Apo SERCA

**Figure 2:**
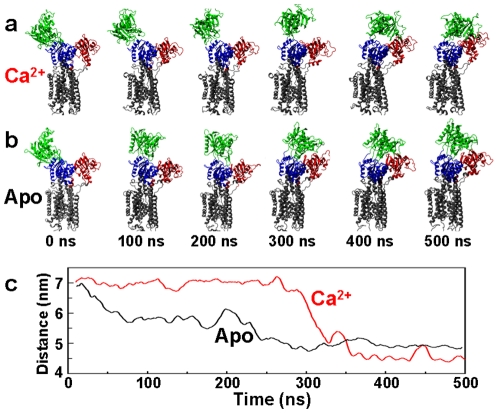
Time-dependent conformational transitions of SERCA. Structural snapshots from the simulations of Ca^2+^-bound (a) and Apo (b) SERCA, showing a complete transition from the open conformation (green N domain and red A domain far apart at 0 ns) to a closed one within 400 ns. (c) C_α_-C_α_ distance between residues Met1 (A domain) and Lys515 (N domain) of Apo (**black**) and Ca^2+^-bound (**red**) SERCA.

We performed two independent 500-ns MD simulations of SERCA (Ca^2+^ and Apo), starting from the crystal structure of the open conformation (1su4, obtained with Ca^2+^ bound to the SERCA transport sites). This crystal structure was chosen for two reasons: (a) because experimental evidence indicates that changes in protonation of SERCA, and not Ca^2+^ binding, drive the transition from E2 to E1 states [19], and (b) because this structure provides a template for simulating SERCA in the open E1 state, which is crucial for elucidating the role of Ca^2+^ in SERCA activation. As described in Methods, the protein was embedded in a lipid bilayer and all atoms were included in the simulation, and the only difference between the two simulation procedures was that Ca^2+^ was removed from the Apo system. **Fig. 2** (a, b) shows snapshots of each system (Ca^2+^ and Apo), following a 10-ns equilibration period. Both simulations show substantial rearrangements of the cytosolic headpiece, producing conformations that are substantially different from that observed in the crystal structure of open SERCA (**Fig. 1**, left). Indeed, both in the presence and absence of Ca^2+^, the final structures observed (after 400 ns) are quite closed (**Fig. 2**a and b), resembling closely the crystal structure obtained in the presence of Ca^2+^ and nucleotide (**Fig. 1**, right). This result helps to reconcile the crystallography and FRET data, since it supports the crystallography-based model that a large-scale open-to-closed structural transition can occur (**Fig. 1**), while supporting the FRET-based conclusion that this structural transition is loosely coupled to biochemical states (defined by bound ligands), since it does not require nucleotide and can occur in both the presence and absence of Ca^2+^ [16]. Indeed, it also supports the FRET observation that the closed conformation of SERCA is the most stable one, even in the presence of Ca^2+^; the open crystal structure must represent a local energy minimum in solution that is trapped by crystal conditions [16].

To analyze these conformational transitions more quantitatively, we plotted the interdomain C_α_-C_α_ distance between residues Met1 (A domain) and Lys515 (N domain) (**Fig. 2c**). We used this distance as a reference to compare our simulations directly with data from FRET experiments with probes attached to these sites [16]. The average simulated distances for the Ca^2+^-bound and Apo SERCA in the final (closed, E2) state are 4.5 and 4.9 nm, respectively, in close agreement with interdomain distances measured experimentally at equilibrium by FRET [16]. The interdomain distance trajectories (**Fig. 2c**) show that the open-to-closed (E1-to-E2) transition occurs much more slowly in the presence of Ca^2+^: Ca^2+^-bound SERCA can sustain the open conformation for 300 ns (**Fig. 2c**, red), while Apo SERCA populates a partially closed headpiece conformation in less than 100 ns, and fully converges to a closed conformation at ∼275 ns (**Fig. 2a**, black). Thus our simulations support the conclusion from the FRET study that the open E1 conformation is not predominant in either biochemical state (Ca^2+^ or Apo) but that E1 is significantly more stabilized in the presence of Ca^2+^. [16]. The agreement between simulated trajectories and experimental observations indicate that the MD simulations provide an accurate picture of the effect of Ca^2+^ on the conformational transitions of SERCA.

Upon closure, the interdomain distance remains fairly stable (ΔR≤0.1 nm), except for one brief interval in the trajectory of Ca^2+^-bound SERCA, where a distance increase of ∼0.5 nm, producing the same distance as in the Apo case, was observed between 429 and 460 ns (**Fig. 2a**, black trace). This temporary increase in interdomain distance results from a separation of the N and A domains (**Fig. S1**, supplementary information), suggesting that discrete closed-to-open transitions of the cytosolic headpiece of Ca^2+^-bound SERCA can occur on the nanosecond time scale.

### Dynamics of individual domains

**Figure 3:**
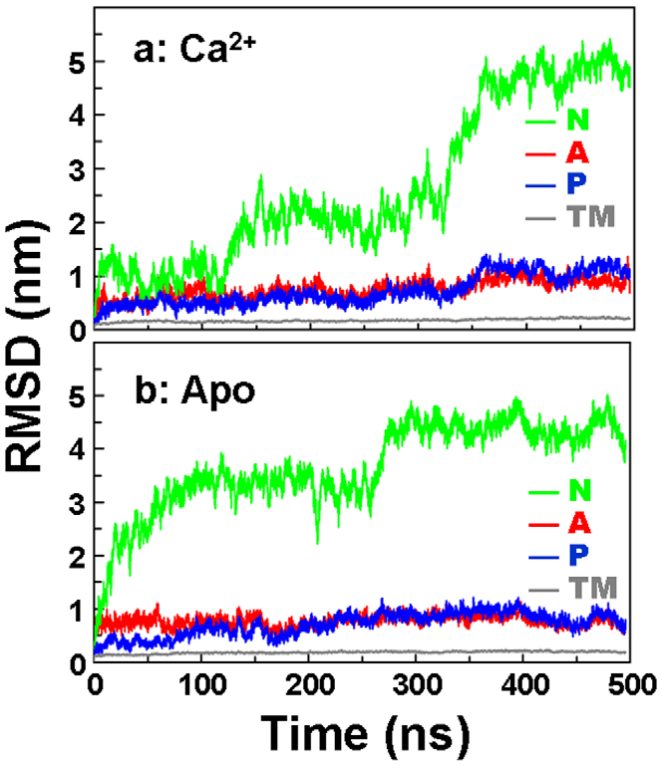
Backbone RMSD of each functional domain of SERCA. Domains are color-coded as indicated, as in Fig. 1. Backbone alignment used the TM domain as a reference.

To evaluate the domain dynamics in more detail, we calculated the backbone root-mean-square deviations (RMSD) for each functional domain of SERCA (**Fig. 3**). In both systems (Ca^2+^ and Apo), the RMSD of the TM domain is very small, with maximum values of 0.23 nm, indicating a clear lack of large-scale structural changes in the sub-microsecond time scale. In contrast, all three domains of the cytosolic headpiece (N, P, and A) show large changes in RMSD relative to the initial structure in both trajectories (**Fig. 3**). The most dramatic changes were observed for the N domain, with maximum RMSD values of 5.0 nm and 4.5 nm for Ca^2+^-bound and Apo SERCA, respectively, and the ns-time-scale fluctuations in RMSD (indicative the domain's instrinsic dynamics) are clearly greater for this domain than for others. The RMSD time course for the N domain (**Fig. 3**) follows a similar pattern to that observed for the N-to-A domain distance in **Fig. 2c**: conformational rearrangement proceeds more slowly in the presence of bound Ca^2+^. In Apo SERCA, the RMSD increases rapidly, reaching a level of 3.5 nm within 100 ns (**Fig. 3b**), while in Ca^2+^-bound SERCA, RMSD remains around 1 nm for over 100 ns and does not exceed 2.5 nm until after 300 ns (**Fig. 3a**), indicating that Ca^2+^ stabilizes the open conformation.

### Spatial arrangement of the cytosolic headpiece in the closed conformation

**Figure 4:**
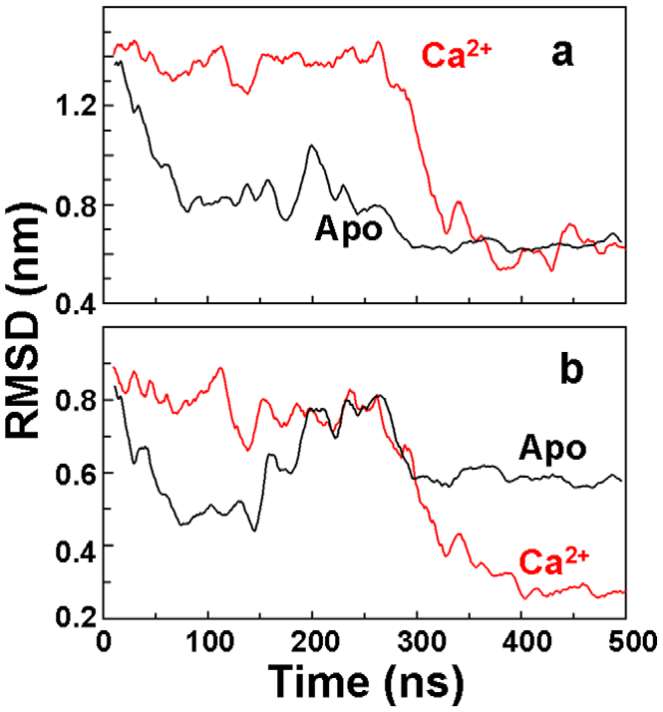
Geometrical rearrangement of the cytosolic headpiece of SERCA. (**a**) RMSD of the entire headpiece of Ca^2+^-bound (**red**) and Apo (black) SERCA; (**b**) RMSD calculated for the N and P domains only in the trajectories of Ca^2+^-bound (red) and Apo (**black**) SERCA. RMSD was calculated by superimposing the backbone atoms of domains N, P and A or N and P, respectively, onto the closed crystal structure of SERCA bound to Ca^2+^ and AMPPCP (PDB: 1vfp). Low RMSD values indicate similarity to the crystal structure.

We have shown above that MD simulations help reconcile previous crystallographic and FRET experiments, indicating that the open-to-closed (E1-to-E2) structural transition is only loosely coupled to the biochemical transitions of ligand binding. The simulated trajectories appear to show that a complete open-to-closed transition is observed whether or not Ca^2+^ is bound to SERCA, even in the absence of ATP (**Fig. 2c**). This leaves open a troubling puzzle: How can such a loosely coupled system result in efficient activation of the enzyme, so that the ATPase reaction is tightly coupled to Ca^2+^ transport? The answer is revealed upon closer inspection of structures observed in the stable phase in the final 100 ns of each trajectory: there is a clear Ca^2+^-dependent difference in the arrangement of the closed headpiece (**Fig. 2a,b**). It is instructive to compare these distinct conformations with the structure of the closed conformation that has been obtained from x-ray crystallography [15]. We performed a quantitative comparison of the spatial arrangement of the headpiece between the MD-generated trajectories and the closed crystal structure of SERCA bound to Ca^2+^ and AMPPCP (**Fig. 4**). We observed that, for both Ca^2+^ and Apo cases, the final simulated arrangement of the entire headpiece differs substantially from that observed in the crystal structure (**Fig. 4a**, RMSD∼0.7 nm). However, when this analysis is restricted to the N and P domains, a clear Ca^2+^-dependant difference is revealed: only in the presence of Ca^2+^ do the N and P domains display an arrangement very similar to that in the closed crystal structure (**Fig. 4b**, RMSD∼0.3 nm).

**Figure 5:**
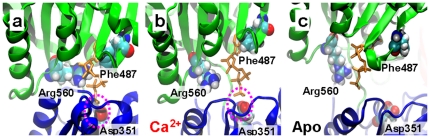
Active site geometry of SERCA in atomic detail. (**a**) Crystal structure of SERCA bound to calcium and AMPPCP (orange sticks). Simulations at 500 ns of (**b**) Ca^2+^-bound and (**c**) Apo SERCA. In both cases, the predicted orientation of AMPPCP was obtained by using rigid-body docking simulations. Residues that are necessary for nucleotide binding (Phe487 and Arg560) and autophosphorylation (Asp351) are shown as van der Waals spheres. The N and P domains are rendered as green and blue ribbons, respectively. The dashed magenta ovals highlight the proximity between the γ-phosphate of AMPPCP and the carboxyl group of Asp351 in **a** and **b**, but not in **c**.

The importance of this difference is illustrated in **Fig. 5**: In the closed crystal structure of SERCA bound to Ca^2+^ and AMPPCP (**Fig. 5a**), the nucleotide is anchored to the N domain by residues Phe487 and Arg560 [15], [20]. These interactions, together with the arrangement of the N and P domains, allow ATP to reach a geometry suitable for γ-phosphate transfer to residue Asp351 (**Fig. 5a**). Unexpectedly, the docking complex between AMPPCP and Ca^2+^-bound SERCA at *t* = 500 ns shows that the nucleotide is oriented in the active site in a similar way as in the crystal structure, with the γ-phosphate in close proximity to the carboxylic group of Asp351 (**Fig. 5b**). The simulated distance between the γ-phosphate of AMPPCP and the carboxylic group of Asp351 of Ca^2+^-bound SERCA is 0.4 nm (**Fig. 5b**), which is identical to that observed in the crystal structure (**Fig. 5a**). In contrast, at the end of the trajectory of Apo SERCA, the γ-phosphate of AMPPCP is located far from Asp351, with a distance of 1.4 nm between the γ-phosphate of AMPPCP and the carboxylic group of Asp351 (**Fig. 5c**). Because the MD simulations were performed in the absence of nucleotide, these results indicate that Ca^2+^ is both necessary and sufficient to generate a headpiece conformation suitable for γ-phosphate transfer; ATP is not required.

The results also showed that ionization of the acidic residues in the Ca^2+^-binding site, which is necessary for the E2 to E1 transition of SERCA, does not favor the formation of the active conformation, indicating that this conformation is unlikely to preexist in solution in the absence of Ca^2+^. In addition, we found that although a closed headpiece conformation can exist in solution (as reported in [16]), this conformation is substantially different compared to that induced by Ca^2+^ (**Fig. 4b**). Together, these observations indicate that Ca^2+^ activates SERCA via an induced-fit mechanism.

While the Ca^2+^-dependent simulation moves the N and P domains into a position similar to that of the closed crystal structure, this is not true for the A domain (**Fig. S2**, Supplementary Information). The arrangement of the A domain observed in the crystal structure has been proposed to lock the cytoplasmic gate of the transmembrane Ca^2+^-binding sites [15]. It is likely that a full convergence to the orientation of the A domain, as observed in the crystal structure, occurs more slowly (i.e., in the microsecond time scale or slower).

## Discussion

We have shown in unprecedented atomic detail, in MD simulations of unprecedented length, that (a) the large-scale open-to-closed structural transition in SERCA is only loosely coupled to the biochemical transitions defined by the binding of ligands (Ca^2+^ and ATP), but (b) only in the presence of Ca^2+^ is SERCA capable of reaching an active conformation via an induced-fit mechanism that positions the N and P domains in a conformation that can facilitate γ-phosphate transfer from ATP to Asp351 (**Fig. 5**). Thus tight coupling of Ca^2+^ activation and ATP hydrolysis is assured. Although full convergence cannot be rigorously demonstrated from a single trajectory, our simulations are far longer than any previous ones in this system, the trajectories appear to be stable over the last 150 ns (**Fig. 2c**, **Fig. 3**), and they are in good agreement with complementary FRET measurements. Thus these simulations appear to capture essential features of the intrinsic dynamics of SERCA activation.

### Free energy landscapes from principal component analysis

**Figure 6:**
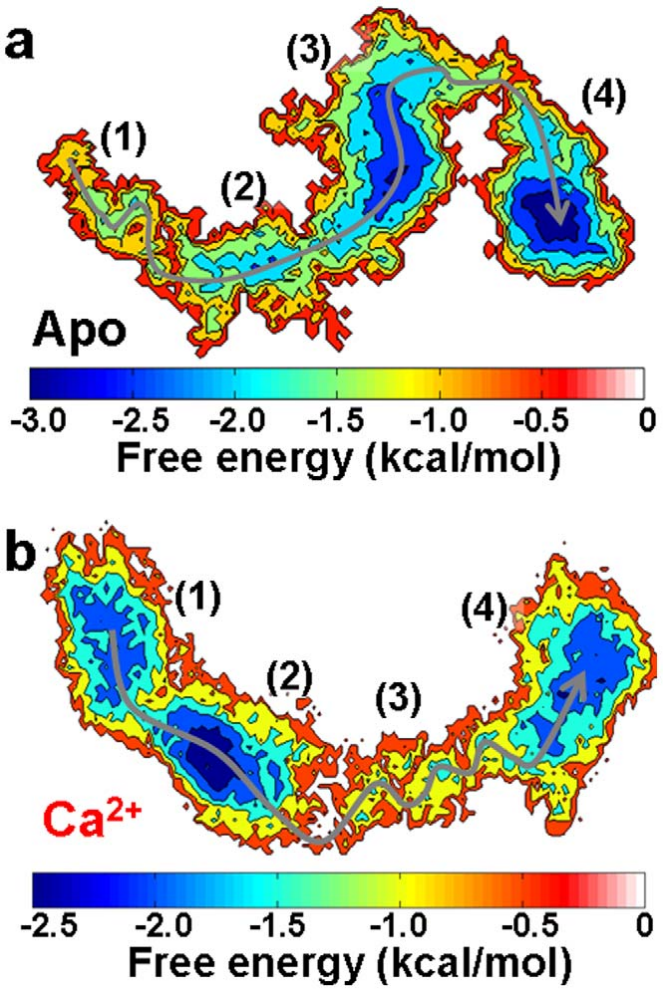
PCA-based free energy landscape of the open-to-closed conformational transition of SERCA. (**a**) Apo SERCA. (**b**) Ca^2+^-bound SERCA. The x and y axes correspond to the the first and second principal components, respectively. The gray arrow shows the preferred direction along the open-to-closed transition in the trajectories. Relevant regions discussed in the text are indicated by numbers in parentheses.

To further connect the structural information with thermodynamics of the open-to-closed transition, we constructed free energy landscapes from the MD trajectories (**Fig. 6**), performing Cartesian principal component analysis (PCA) using CARMA [21]. PCA uses the actual dynamics of the protein to generate the appropriate collective coordinates that capture the important motions of the native states of the protein [22], [23]. In Apo SERCA, the open conformation is thermodynamically unfavorable (region 1, **Fig. 6a**) and therefore relaxes rapidly to a partially closed conformation. The evolution from a partially closed conformation (regions 2 and 3) to a closed conformation (region 4) occurs linearly with a small energy barrier, explaining the fast kinetics of the open-to-closed transition (**Fig. 2a**).

**Figure 7:**
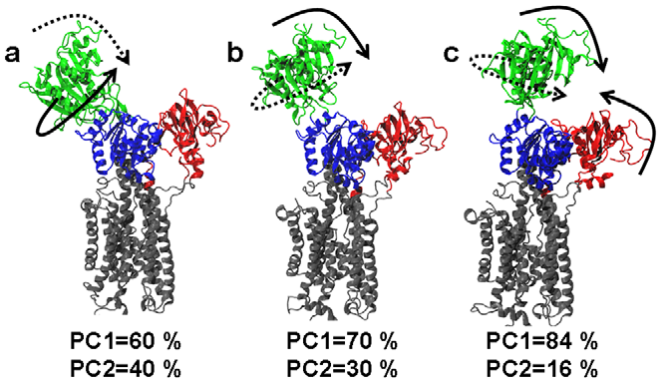
Directionality of motions along the open-to-closed transition of Ca^2+^-bound SERCA. The motions correspond to (a) region 1, (b) region 2 and (c) region 3 of the energy landscape (Fig. 6B). Dashed and solid arrows represent motions described by the first and second principal components (PC1 and PC2), respectively. The percentage of contribution of each principal component (PC) that belongs to the essential space is shown below each structure.

On the other hand, the energy landscape of Ca^2+^-bound SERCA reveals characteristics that are not observed in Apo SERCA. There are two well-defined minima, 1 and 2 (**Fig. 6b**), connected with each other by a narrow energy channel, both structurally represented by an open headpiece conformation. This observation is intriguing, because from **Fig. 2c** one might expect a single energy minimum during the first 300 ns of simulation. This apparent contradiction can be reconciled by examining the motions of SERCA that are associated with the essential principal components (degrees of freedom) [22]. In both energy minima, the motions represented by these two components are the same: hinge-bending and twisting of the N domain with respect to the P domain (**Fig. 7a,b**). However, in minimum 1, the twisting motion is dominant (**Fig. 7a**), while in minimum 2, the bending motion is dominant (**Fig. 7b**).

Each of the two free energy landscapes constructed here is based on a single 500 ns simulation, so the results should be interpreted with caution – the free energy wells would probably deepen significantly if the simulations were extended. However, the free energy landscape and collective motions combine to suggest that Ca^2+^ splits the ensemble of open conformations into two minima to selectively produce the collective motions of the N domain necessary for the closure of the headpiece toward the active conformation. Considering that SERCA populates open, partially open and closed conformations in solution, and based on comparison of the free energy landscapes of both Apo and Ca^2+^-bound SERCA, we suggest that the open conformation of SERCA belongs to a conformational pathway necessary for the selective activation of the high Ca^2+^ affinity conformation of SERCA by Ca^2+^. This goes beyond previous observations [16], not only suggesting that the open conformation of SERCA can exist in solution, but also that the open conformation is a functional element of the conformational space of the pump.

A particularly important region of the free energy landscape of Ca^2+^-bound SERCA is region 3, which corresponds to the actual open-to-closed transition. Unlike Apo SERCA, this region of the energy landscape is very rugged, and is characterized by multiple local minima separated by small energy barriers (∼0.5 kcal/mol) (**Fig. 7b**). This indicates that Ca^2+^ binding reshapes the free energy landscape to contain several kinetic traps. We suggest that these kinetic traps are an intrinsic feature of Ca^2+^-bound SERCA, and that they act as checkpoints to drive the headpiece through a well-defined pathway toward a correct geometry necessary for γ-phosphate transfer to Asp351. This suggestion also challenges the current view that the orientational distribution of the N domain could represent a random sampling of conformations in solution [24]. While this is probably true for Apo SERCA, our results strongly suggest that in the presence of Ca^2+^ the N domain strictly follows a well-defined pathway between the open and closed conformations (**Fig. 7a–c**). This exquisite malleability of the free energy landscape appears responsible to fine-tune SERCA activity: in Apo SERCA, the energy landscape ensures that ATP will not be used unnecessarily, while in the presence of Ca^2+^ the reshaping of the landscape guarantees that SERCA is not trapped in an unproductive conformation, therefore facilitating ATP hydrolysis and the formation of the phosphoenzyme complex, necessary for calcium transport to the lumen [25].

### Previous MD simulations of SERCA

In a recent paper, MD simulations of Ca^2+^-bound SERCA were reported [26], but these were only 10 ns in duration, similar to the equilibration period in the present study. In these 10-ns simulations, only very small conformational changes were observed, with RMSD less than or equal to 0.7 nm, due primarily to rigid body movements of the N and A domains. Based on our results, these conformational changes correspond to the relaxation of SERCA in solution (as carried out in our 10-ns equilibration step), while functionally significant conformational changes are much larger and occur on a much longer time scale.

### Conclusion

MD simulations revealed a large-scale open-to-closed conformational transition of full-length SERCA in the presence and absence of Ca^2+^. Although this large-scale structural transition natively occurs in SERCA and is not tightly coupled to Ca^2+^ or ATP binding, Ca^2+^ is necessary and sufficient for SERCA to reach the precise geometrical arrangement necessary for ATP hydrolysis. Ca^2+^ plays a crucial role in activating SERCA by reshaping its free energy landscape, which creates a path between the open conformation and the active closed conformation. Based on the observed Ca^2+^-dependent changes in the free energy landscape, we speculate that the allosteric activation of SERCA does not rely on a specific network of interactions between the Ca^2+^-binding site and the headpiece, but on the malleability of the free energy landscape within SERCA. This thermodynamic tuning effectively connects dynamics with function in SERCA, thus ensuring that ATP hydrolysis is tightly coupled to Ca^2+^ transport.

Although X-ray crystallography has proven to be a powerful tool in understanding the atomic structure of SERCA, this study supports the conclusion, based on previous FRET experiments [16], that the open crystal structure represents a local energy minimum that is not predominant in solution. It is clear that solution spectroscopic methods such as FRET, EPR, and NMR are needed to connect real-time protein dynamics with function, resolving transitions among multiple conformational states [8], [9], [27]–[30]. Future spectroscopic studies, coordinated with MD simulations of other steps in the SERCA reaction cycle (e.g., the E2-E1 transition, phosphoenzyme hydrolysis) will be needed to define completely this complex mechanism. In conclusion, these MD simulations demonstrate the importance of real-time dynamics in the formation of catalytically competent conformations of SERCA, with broad implications for the understanding of enzymatic catalysis in atomic detail.

## Methods

### Molecular dynamics simulations

We used the crystal structure of SERCA 1a with two bound Ca^2+^ (PDB: 1su4) as a starting point for the simulations of Ca^2+^-bound and Apo SERCA. For the simulation of Ca^2+^-bound SERCA, we used the crystal structure as is, keeping all ions and water molecules. A similar approach was used to simulate Apo SERCA, except for the removal of Ca^2+^ from the structure; furthermore, we adjusted the ionization states of the acidic residues of Ca^2+^-binding sites to mimic the E1 state of SERCA [19], [31]. We adjusted the pH of the system to a value of ∼7.0 by adjusting side-chain ionization states using PROPKA [32]. Each protein system was inserted in a pre-equilibrated 13×13 nm bilayer of palmitoyl-2-oleoyl-sn-glycerol-phosphatidylcholine (POPC) using VMD [33]. This initial system was further solvated using TIP3P water molecules with a minimum margin of 1.5 nm between the protein and the edges of the periodic box in the *z*-axis. Finally, Na^+^ and Cl^−^ ions were added to the system to neutralize the charge of the system and to produce an ion concentration of approximately 150 mM. Both Ca^2+^-bound and Apo SERCA systems contained ∼270,000 atoms. CHARMM22 force field topologies and parameters with CMAP correction [34], [35] were used for the protein, lipid, water and ions.

MD simulations were performed by using the program NAMD 2.7 [36], with periodic boundary conditions [37], particle mesh Ewald [38], [39], a nonbonded cutoff of 0.9 nm, and a 1 fs time step. The NPT ensemble was maintained with a Langevin thermostat (310K) and an anisotropic Langevin piston barostat (1 atm). The systems were first subjected to energy minimization for 1 ns, followed by gradually warming up of the systems for 200 ps. This procedure was followed by 10 ns of equilibration with backbone atoms harmonically restrained using a force constant of 5.0 kcal mol^−1^ Å^−2^). Each simulation was continued for 500 ns without any restraints.

### Docking simulations

Docking simulations of AMPPCP to the ATP-binding site were performed with AutoDock 4.2 [40]. Grid maps of 2.5×2.5×2.5 nm representing the protein were centered on the ATP-binding site. During docking simulations, both AMPPCP and SERCA were kept rigid; and the conformation of AMPPCP was kept as it appears in the crystal structure of SERCA bound to AMPPCP and Ca^2+^ (PDB: 1vfp). Docking simulations were carried out using the Lamarckian Genetic Algorithm, with an initial population of 100 individuals, a maximum number of 10,000,000 energy evaluations and a maximum number of 50,000 generations. Docking simulations consisted of 100 independent runs. Resulting orientations lying within 0.15 nm in the RMSD were clustered together, and the best-ranked orientation was used for analysis.

## Supporting Information

Figure S1 Spontaneous closed-to-open transitions of Ca^2+^-bound SERCA. The transition was detected in the trajectory between 430 and 450 ns, and it is characterized by an increase in the in C_α_-C_α_ distance between residues Met1 (A domain) and Lys515 (N domain). This distance is shown in the cartoons as a black arrow.
(TIF)

Figure S2 Comparison of the headpiece arrangement between MD simulation and crystal structure of Ca^2+^-bound SERCA. Backbone superimposition of the structure of Ca^2+^-bound SERCA at the end of the simulation (solid ribbons) and the crystal structure of SERCA bound to Ca^2+^ and AMPPCP (transparent ribbons). While N and P domains move into positions similar to those of the crystal structure, this is not true for the A domain. The oval indicates the A-N interdomain contact observed in the crystal structure but not in the MD simulation, and the arrow indicates the direction of the expected movement of the A-domain.
(TIF)
