# Supplementary figures and images for: Atomic-Level Characterization of the Activation Mechanism of SERCA by Calcium
Source: PLoS One. 2011 Oct 27;6(10):e26936. doi: 10.1371/journal.pone.0026936 (PMC3203174; doi:10.1371/journal.pone.0026936)

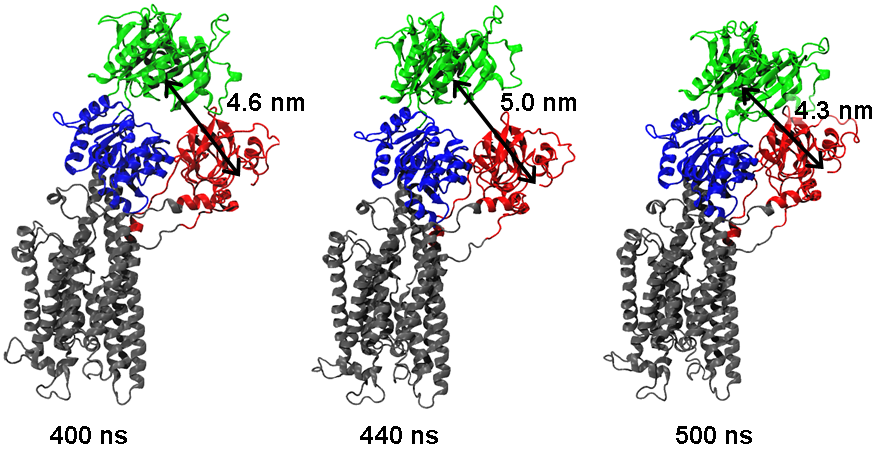

Supplement: Figure S1 — Spontaneous closed-to-open transitions of Ca2+-bound SERCA. The transition was detected in the trajectory between 430 and 450 ns, and it is characterized by an increase in the in Cα-Cα distance between residues Met1 (A domain) and Lys515 (N domain). This distance is shown in the cartoons as a black arrow. (TIF) [file pone.0026936.s001.tif]

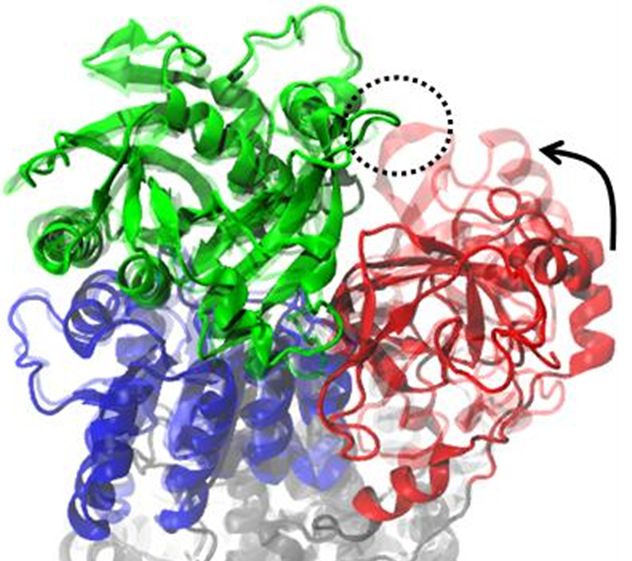

Supplement: Figure S2 — Comparison of the headpiece arrangement between MD simulation and crystal structure of Ca2+-bound SERCA. Backbone superimposition of the structure of Ca2+-bound SERCA at the end of the simulation (solid ribbons) and the crystal structure of SERCA bound to Ca2+ and AMPPCP (transparent ribbons). While N and P domains move into positions similar to those of the crystal structure, this is not true for the A domain. The oval indicates the A-N interdomain contact observed in the crystal structure but not in the MD simulation, and the arrow indicates the direction of the expected movement of the A-domain. (TIF) [file pone.0026936.s002.tif]
